# Transcriptomic Analysis of Leaves from Two Maize Hybrids Under Heat Stress During the Early Generative Stage

**DOI:** 10.3390/genes16050480

**Published:** 2025-04-24

**Authors:** Siqi Zhang, Lei Sun, Chunhong Ma, Dajin Xu, Bo Jiao, Jiao Wang, Fushuang Dong, Fan Yang, Shuo Zhou, Qing Yang, Pu Zhao

**Affiliations:** 1 Institute of Biotechnology and Food Science, Hebei Academy of Agriculture and Forestry Science/Hebei Key Laboratory of Plant Genetic Engineering, Shijiazhuang 050051, China; zhangsiqi202210@163.com (S.Z.); mch0609@126.com (C.M.); 15230833898@163.com (D.X.); jiaobo1206@163.com (B.J.); xiangxiemaoshe@163.com (J.W.); dongfushuang@126.com (F.D.); yf21st@sina.com (F.Y.); zhoushuobio@163.com (S.Z.); 2 College of Agronomy and Biotechnology, Hebei Key Laboratory of Crop Stress Biology, Hebei Normal University of Science and Technology, Qinhuangdao 066600, China

**Keywords:** maize, heat stress, early generative stage, transcriptome

## Abstract

Background: High temperatures during the early generative stage significantly threaten maize productivity, yet the molecular basis of heat tolerance remains unclear. Methods: To elucidate the molecular mechanisms of heat tolerance in maize, two hybrids—ZD309 (heat-tolerant) and XY335 (heat-sensitive)—were selected for integrated transcriptomic and physiological analyses. The plants were subjected to high-temperature treatments (3–5 °C above ambient field temperature) for 0, 1, 3, 5, and 7 days, with controls grown under natural conditions. Physiological indices, including Superoxide dismutase (SOD) activity, and proline (PRO), malondialdehyde (MDA), soluble sugar, and protein content, were measured. Results: Transcriptome analysis identified 1595 differentially expressed genes (DEGs) in XY335 (509 up- and 1086 down-regulated) and 1526 DEGs in ZD309 (863 up- and 663 down-regulated), with the most pronounced changes occurring on day 5. Key DEGs in XY335 were enriched in galactose metabolism and carbohydrate catabolism, whereas ZD309 exhibited rapid activation of oxidative stress and cell wall integrity pathways. Mfuzz time-series analysis categorized DEGs from XY335 and ZD309 into six clusters each. Weighted gene co-expression network analysis (WGCNA) identified 10 hub genes involved in ubiquitin thioesterase activity and RNA modification, suggesting protein-level regulatory roles. Conclusions: This study reveals distinct transcriptional dynamics between heat-tolerant and heat-sensitive varieties, providing candidate genes for breeding thermotolerant maize and advancing our understanding of heat stress responses during critical reproductive stages.

## 1. Introduction

Maize, together with rice and wheat, ranks among the world’s three most significant cereal crops and holds a crucial role in global agriculture [1]. Heat stress represents a major abiotic constraint on global maize productivity, with elevated temperatures causing significant yield reductions across multiple maize-producing regions worldwide [2,3,4,5]. In recent years, the main producing areas of summer maize in China have frequently been afflicted by high temperatures and extremely high temperatures during the flowering period, with most places seeing temperatures of more than 35 °C, and some even exceeding 40 °C [5]. The high temperature during the flowering stage had the largest effect on the number of grains per ear and the highest decrease in yield [6]. High temperatures during the flowering period can reduce the leaf area index and photosynthetic pigment content of summer maize. The activity of ribulose-1,5-bisphosphate carboxylase (RuBP carboxylase) and phosphoenolpyruvate carboxylase (PEP carboxylase) in the leaves declined, and the photosynthetic performance was inhibited. As a result, the accumulation and distribution of photosynthetic assimilations were blocked, the number of grains per ear decreased, and the grain weight and yield reduced [7].

Multiple molecular mechanisms regulating maize thermotolerance have been identified. Notably, heat stress induces oxidative damage through reactive oxygen species (ROS) overaccumulation [8]. The intracellular ROS concentration in plants is precisely regulated by antioxidant scavenging systems to maintain ROS homeostasis, demonstrating a positive correlation with maize thermotolerance [9]. Genes associated with metabolic processes, including protein homeostasis, endoplasmic reticulum function, and phytohormone signaling pathways, may constitute critical regulatory elements in heat stress responses [10,11]. Furthermore, heat shock proteins (HSPs), functioning as molecular chaperones, participate in diverse cellular processes, particularly in thermotolerance mechanisms [12]. Heat stress represents a major abiotic constraint on global maize productivity, with elevated temperatures causing significant yield reductions across multiple maize-producing regions worldwide [13]. The heat shock transcription factor (HSF) is a critical transcription factor in plant heat stress responses which respond to heat stress and regulate downstream genes’ expression, thereby helping plants adapt to high temperature. *ZmHSF20* negatively regulates heat tolerance by inhibiting the downstream gene expression of cellulose synthetase gene *ZmCesA2* and heat shock transcription factor *ZmHsf4* [14]. Furthermore, calcium-dependent protein kinase (CDPK) serves as a pivotal regulatory enzyme mediating multiple physiological processes in plants, particularly during abiotic stress responses. Specifically, *ZmCDPK7* enhances maize thermotolerance through phosphorylation-mediated activation of cytosolic heat shock proteins’ chaperone activity. Additionally, *ZmCDPK7* modulates the expression of critical redox-related enzymes, including respiratory burst oxidase homologs (*RBOHs*), catalase 1 (*CAT1*), and ascorbate peroxidase 1 (*APX1*), thereby orchestrating ROS homeostasis under high-temperature-stress conditions [15].

Heat stress during the reproductive stage adversely impacts maize yield components, including ear architecture, kernel weight, kernel number per ear, and overall grain productivity [16,17,18]. However, when high-temperature stress persists for prolonged durations or exceeds a certain intensity threshold, it results in ROS accumulation. This, in turn, triggers membrane lipid peroxidation, damaging the cell structure and function. In addition, under high-temperature stress during the flowering period, through transcriptomic analysis, a large number of important genes related to the heat-tolerance response mechanism in maize have been discovered. These genes mediate critical pathways such as antioxidant redox, and maintain the cell homeostasis and physiological processes of maize under high-temperature stress. Metabolomic analysis also indicated that during the response to heat stress of maize, significant changes occurred in the products related to lipids, flavonoids, and energy metabolism, revealing its complex metabolic regulation mechanism [19].

Integrated analysis of RNA sequencing (RNA-seq) and WGCNA have emerged as powerful and efficient approaches for identifying functionally relevant candidate genes and regulatory networks associated with stress responses. WGCNA is mainly used to study the biological relationship between co-expression gene modules and target traits, and to identify core genes in the co-expression network. As a representative systems biology method, WGCNA has been widely applied in plant research [20,21]. For example, through WGCNA analysis, corresponding core genes were found in plants such as rice [22], cotton [23], corn [24].

The frequent occurrence of high temperatures has become a major factor influencing maize productivity. The elucidation of the high-temperature tolerance mechanisms in maize is a crucial aspect of resistance breeding. The current research on high-temperature tolerance in maize primarily focuses on the seedling stage. In this study, ZD309 and XY335, two commercial varieties, were used to examine the effects of elevated temperature exposure during the flowering stage on maize. Transcriptome analysis was employed to examine the changes in regulatory networks of ZD309 and XY335 at 0, 1, 3, 5, and 7 days after high-temperature stress. The results of this research contribute to a better understanding of the molecular mechanisms underlying heat tolerance in maize and provide valuable genes for targeted breeding improvement.

## 2. Materials and Methods

### 2.1. Plant Materials and Heat Treatments

The heat-tolerant maize variety ZD309 and the heat-sensitive maize variety XY335 were provided by the Hebei Academy of Agriculture and Forestry and were cultivated on experimental farmland at the Hebei Academy of Agriculture and Forestry Sciences (Shijiazhuang, Hebei, China; 38°07′ N, 114°22′ E). Field-based high-temperature treatments were conducted using custom-built growth chambers (3 m × 4 m × 4 m, L × W × H). The sides were enclosed with 95% light-transmittance resin film, and the top was 90% sealed with a 10% gap for gas exchange. Heat treatment was applied starting from the flowering stage, with the temperature maintained 3–5 °C higher than the field temperature. The continuous-high-temperature treatment was conducted for 7 days, with a day and night temperature controlled at 43/26 °C (day/night). During the treatment period, the average day and night temperature under natural conditions was 39/23 °C (day/night). Canopy temperature and relative humidity were monitored via a thermohygrometer. Maize plants grown under natural conditions outside the shed were used as the control. The growth box was removed at the end of the heat treatment to allow the maize plants to grow under natural conditions, and the experiment was replicated three times. During the high-temperature treatment, the relative humidity and other growth conditions were maintained at levels roughly equivalent to those outside the growth chamber. Ear-leaf samples (three replicates) were harvested at 0, 1, 3, 5, and 7 days after heat treatment for each variety, immediately frozen in liquid nitrogen and stored at −80 °C.

### 2.2. Measurement of Heat-Stress-Related Physiological Indexes

Physiological analyses were performed using ear-leaf samples from ZD309 and XY335. SOD activity, along with the contents of PRO, MDA, soluble protein, and soluble sugar, were quantified using commercial assay kits (SOD-1-W, PRO-1-Y, MDA-1-Y, BCAP-1W, KT-1-Y; Suzhou Comin Biotechnology Co., Ltd., Suzhou, China) according to the manufacturer’s protocols.

### 2.3. RNA Library Construction and Sequencing

Total RNA was extracted from ear-leaves by using Trizol Reagent (Invitrogen Life Technologies, Carlsbad, CA, USA). The RNA quality was detected by a NanoDrop sepectrophotometer (Thermo Scientific, Waltham, MA, USA). The RNA quality assessment was performed using a NanoDrop spectrophotometer (Thermo Fisher Scientific, Waltham, MA, USA). A first-strand cDNA synthesis was carried out using HiScript III RT SuperMix for qPCR (+gDNA wiper) (Vazyme Biotech, Nanjing, China), a commercial reverse transcription system featuring integrated genomic DNA elimination to prevent contamination. Following cDNA synthesis, PCR amplification was performed for library construction. The resulting cDNA libraries were purified using the AMPure XP system (Beckman Coulter, Beverly, MA, USA), followed by quality assessment and quantification using an Agilent 2100 Bioanalyzer (Agilent Technologies, Santa Clara, CA, USA). Final sequencing was conducted on an Illumina NovaSeq 6000 platform (Personal Biotechnology Co., Ltd., Shanghai, China) with 150 bp paired-end reads. All experimental procedures were performed in triplicate to ensure technical reproducibility.

### 2.4. Transcriptome Analysis

To efficiently and accurately analyze the sequencing results, the raw reads were filtered by deleting low-quality reads (reads in which the low quality (Q ≤ 5) base number > 50%). Quality-filtered reads from each library were then aligned to the maize B73 reference genome (B73 RefGen_v4; MaizeGDB) using HISAT2 (version 2.2.1) with default parameters [25]. Gene-level read counts were quantified using featureCounts (v2.0.1) with default parameters, assigning reads to genomic features based on the B73 RefGen_v4 annotation [26]. DEGs were identified using DESeq2 (v1.34.0) with a significance threshold of |log2 fold change| ≥ 1 and a false discovery rate (FDR) < 0.05 after Benjamini–Hochberg correction [27]. Functional annotation of DEGs was performed through Gene Ontology (GO) and Kyoto Encyclopedia of Genes and Genomes (KEGG) pathway analyses. Enrichment analysis and visualization were conducted using the ClusterProfiler package (v4.6.0) in R, implementing the WGCNA framework for network-based functional characterization [28]. KEGG pathway enrichment analysis was conducted using ClusterProfiler (v4.6.0), with significantly enriched pathways identified at a threshold of adjusted *p*-value < 0.05 (Benjamini–Hochberg correction). Transcription factor (TF) prediction among DEGs was performed using ITAK software (http://bioinfo.bti.cornell.edu/tool/itak, accessed on 17 March 2025) [29], which integrates the PlnTFDB3.0 (http://plntfdb.bio.uni-potsdam.de/v3.0/, accessed on 17 March 2025) [30] and PlantTFDB3.0 (http://planttfdb.cbi.pku.edu.cn, accessed on 17 March 2025) [31] databases.

### 2.5. Quantitative Real-Time PCR

To validate the RNA-seq results, the expression levels of six randomly selected DEGs were assessed by quantitative reverse transcription PCR (qRT-PCR) using the original RNA samples. Gene-specific primers were designed using NCBI Primer-BLAST, ensuring amplification specificity. Total RNA from RNA-seq samples was reverse-transcribed using PrimeScript™ RT Reagent Kit with gDNA Eraser (Takara, Dalian, China). qRT-PCR analysis was conducted using the 7500 Fast Real-Time PCR System (Applied Biosystems, Foster, CA, USA) with TransStart Top Green qPCR SuperMix (TransGen Biotech, Beijing, China). All reactions included three technical replicates per sample. Relative gene expression levels were calculated using the 2^−∆∆Ct^ method, with the *ACT_2_* serving as the endogenous reference gene.

### 2.6. Statistical Analysis

All physiological parameters and qRT-PCR-derived gene expression data were analyzed using one-way analysis of variance (ANOVA) and Student’s *t*-test in SPSS 22.0 (IBM Corporation, New York, NY, USA). Statistical significance thresholds were defined as *p* < 0.05 for significant differences and *p* < 0.01 for highly significant differences.

## 3. Results

### 3.1. Analysis of Physiological Indicators Under High-Temperature Stress

**Figure 1:**
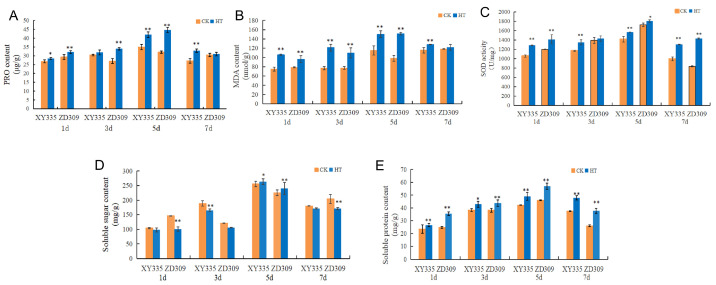
Effect of high-temperature stress on the physiological parameters of maize in ZD309 and XY335. (**A**) SOD activity was expressed in U mg^−1^ (units per milligram), representing enzymatic activity per milligram of fresh leaf tissue. (**B**) PRO content was quantified in μg g^−1^ (micrograms per gram), reflecting PRO accumulation per gram of fresh weight. (**C**) MDA concentration was measured in nmol g^−1^ (nanomoles per gram), indicating lipid peroxidation levels per gram of fresh tissue. (**D**,**E**) Soluble sugar and soluble protein contents were determined in mg g^−1^ (milligrams per gram), representing their respective concentrations per gram of fresh biomass. * and ** indicate that the corresponding physical character in heat-stressed maize plants exhibit significantly and very significantly different compared to the control at *p* < 0.05 and *p* < 0.01 levels, respectively.

To assess high-temperature-stress effects on physiological parameters, SOD activity, along with PRO, MDA, soluble sugar, and soluble protein contents in maize ear-leaves under heat stress were quantified. The results showed that the activity of SOD, the contents of PRO, MDA and soluble protein increased significantly under heat stress. In ZD309 and XY335, all of the physiological parameters were significantly increased on the 5th day (Figure 1).

### 3.2. Transcriptome Analysis of Flowering Stage in Maize Hybrids Under Heat Stress

High-throughput sequencing was performed on maize leaves treated at different times. Sequencing of 30 samples generated 143.78 GB of raw data, which yielded 132.54 GB of high-quality reads following low-quality read filtration. The clean datasets exhibited Q30 scores (base call accuracy ≥ 99.9%) ranging from 93.53% to 94.26%, indicating superior base-calling accuracy. Alignment against the reference genome revealed unique mapping rates of 95.38–96.17% across samples.

### 3.3. Identification of DEGs for XY335 and ZD309 Under Heat Stress During Flowering Stage

**Figure 2:**
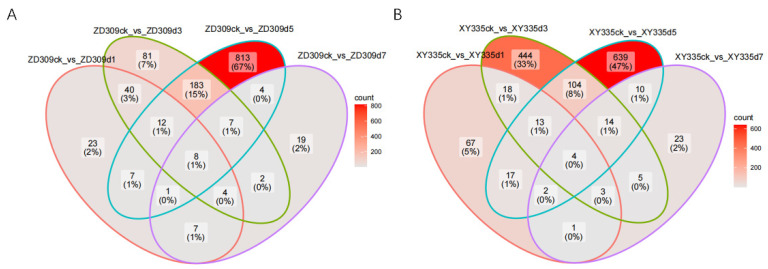
Analysis of DEGs in ZD309 and XY335 with different treatment days under heat stress. (**A**) DEGs in ZD309. (**B**) DEGs in XY335.

**Figure A1:**
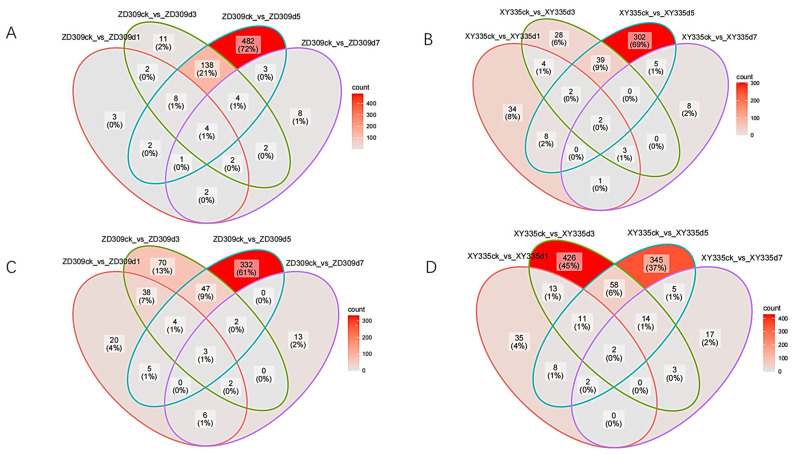
Analysis of all up- and down-regulated DEGs of ZD309 and XY335 under heat stress. (**A**) DEGs up-regulated by ZD309. (**B**) DEGs up-regulated by XY335. (**C**) DEGs down-regulated by ZD309. (**D**) DEGs down-regulated by XY335.

Transcriptomic analysis identified 1595 DEGs in *XY335*, comprising 509 up-regulated and 1086 down-regulated transcripts. In contrast, *ZD309* exhibited 1526 DEGs, with 863 transcripts showing up-regulation and 663 demonstrating down-regulation under comparable experimental conditions (Figure 2 and Figure A1). Compared with the treatment of different time duration, most of the DEGs were identified on the 5th day in both ZD309 and XY335. In ZD309, under high-temperature stress, the comparison groups ZD309CK vs. ZD309D1, ZD309CK vs. ZD309D3, ZD309CK vs. ZD309D5, and ZD309CK vs. ZD309D7 contained 23, 81, 813, and 19 unique DEGs, respectively. Eight DEGs were consistently identified in each of the four comparison groups (Figure 2A). In XY335, the comparison groups XY335CK vs. XY335D1, XY335 CK vs. XY335D3, XY335 CK vs. XY335D5, and XY335 CK vs. XY335D7 showed 67, 444, 639, and 23 unique DEGs, respectively, with four DEGs shared in each of the four groups (Figure 2B). Compared to ZD309, XY335 exhibited a larger number of unique DEGs. Analysis of DEGs revealed that in ZD309, the up-regulated DEGs had 3, 11, 482, and 8 unique DEGs in each comparison group, respectively, while the down-regulated DEGs had 20, 70, 332, and 13 unique DEGs in each comparison group, respectively. In XY335, the up-regulated DEGs that were unique in each comparison group were 34, 28, 302, and 8, respectively, while the down-regulated DEGs had 35, 426, 345, and 17 unique DEGs in each comparison group, respectively (Figure A1). In ZD309, the up-regulated DEGs outnumbered down-regulated ones, whereas XY335 exhibited the inverse pattern, with down-regulated DEGs predominating. The number of DEGs in XY335 and ZD309 after 3 and 5 days of high-temperature treatment was higher than that observed on the first day, suggesting that prolonged exposure to heat stress enabled these varieties to initiate adaptive responses over time.

**Figure 3:**
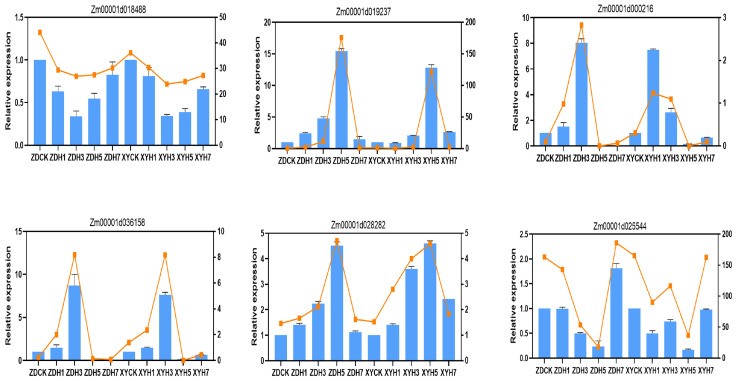
Verification of DEGs by qRT-PCR. Expression profiles of six randomly selected genes were quantified using the 2^−∆∆Ct^ method. Blue bar graphs represent qRT-PCR results, with error bars indicating mean values ± standard error of the mean (SEM). Orange trendlines illustrate corresponding normalized RNA-seq expression levels (FPKM values) for each sample. The experiment was conducted with five biological replications.

To confirm the reliability of the RNA-seq results, six randomly selected DEGs were subjected to expression validation through qRT-PCR. The qRT-PCR-derived expression profiles showed concordance with the transcriptomic data, corroborating the RNA-seq findings (Figure 3).

**Figure 4:**
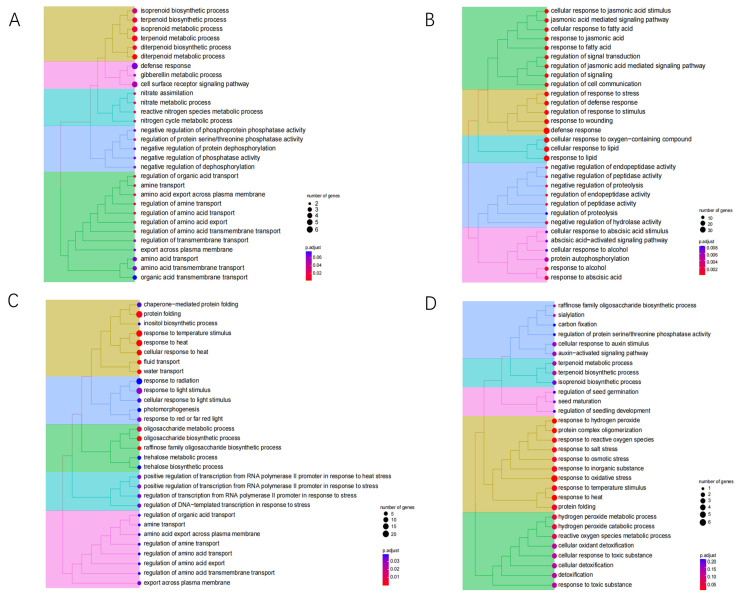
GO analysis of XY335 DEGs under heat stress. (**A**) GO enrichment of XY335 after high-temperature treatment for one day. (**B**) GO enrichment of XY335 after high-temperature treatment for three days. (**C**) GO enrichment of XY335 after high-temperature treatment for five days. (**D**) GO enrichment of XY335 after high-temperature treatment for seven days.

**Figure 5:**
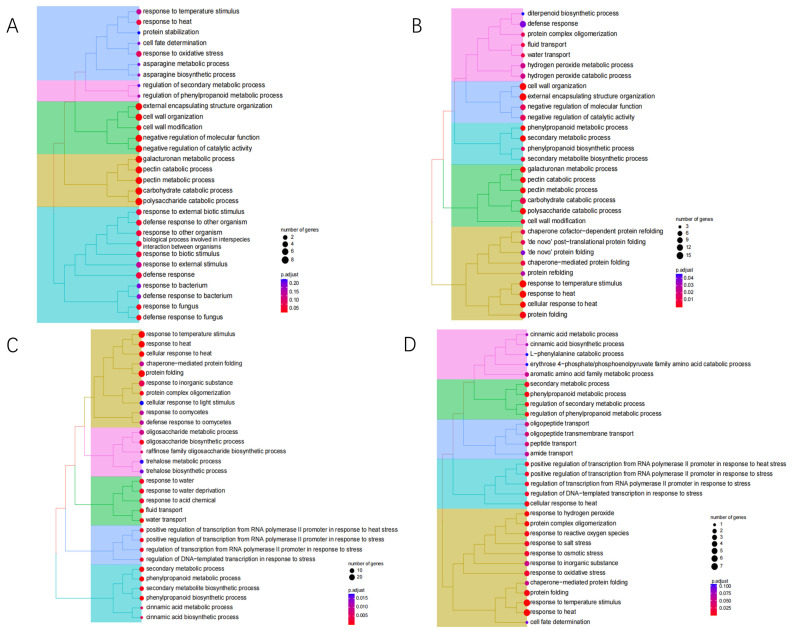
GO analysis of ZD309 DEGs under heat stress. (**A**) GO enrichment of ZD309 after high-temperature treatment for one day. (**B**) GO enrichment of ZD309 after high-temperature treatment for three days. (**C**) GO enrichment of ZD309 after high-temperature treatment for five days. (**D**) GO enrichment of ZD309 after high-temperature treatment for seven days.

**Figure 6:**
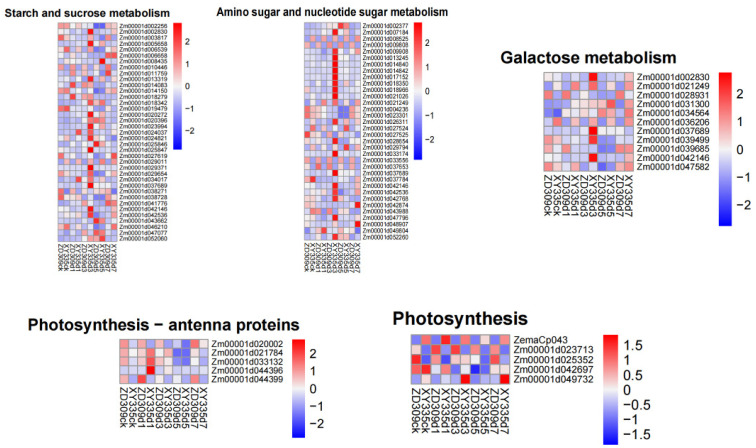
The primary metabolic pathways analysis of DEGs in XY335 and ZD309 under heat stress using Z-score.

To investigate the molecular responses to heat stress, the DEGs in cultivars XY335 and ZD309 were subjected to GO and KEGG pathway enrichment analyses. For XY335 under short-term heat stress (1 day), DEGs were predominantly enriched in the terpene biosynthesis pathway (Figure 4A). Extended heat exposure revealed temporal-specific responses: After 3 days of treatment, significant enrichment shifted to defense-related processes including the jasmonate-mediated signaling pathway, cellular response to fatty acids, and general defense mechanisms (Figure 4B). By day 5, DEGs showed primary association with protein homeostasis maintenance through protein-folding pathways, coupled with temperature response regulation and oligosaccharide metabolic processes (Figure 4C). Prolonged 7-day heat stress triggered oxidative stress responses, with predominant enrichment in hydrogen peroxide and ROS response pathways (Figure 4D). In contrast, ZD309 exhibited distinct temporal patterns. Early response (day 1) primarily involved carbohydrate catabolism activation (Figure 5A). Both 3- and 5-day treatments consistently engaged protein-folding pathways and secondary metabolite biosynthesis (Figure 5B,C). Similar to XY335, the 7-day treatment induced oxidative stress responses alongside continued protein-folding processes (Figure 5D). KEGG pathway analysis identified several key pathways significantly impacted by high-temperature stress, including amino acid/nucleotide metabolism, starch/sucrose metabolism, fatty acid biosynthesis, photosynthesis-related pathways (antenna proteins and core processes), and galactose metabolism. Notably, the most pronounced pathway alterations across both cultivars occurred in XY335 at the 3-day treatment timepoint (Figure 6), suggesting this phase represents a critical transition period in thermotolerance mechanisms.

### 3.4. Mfuzz Time-Series Analysis

**Figure 7:**
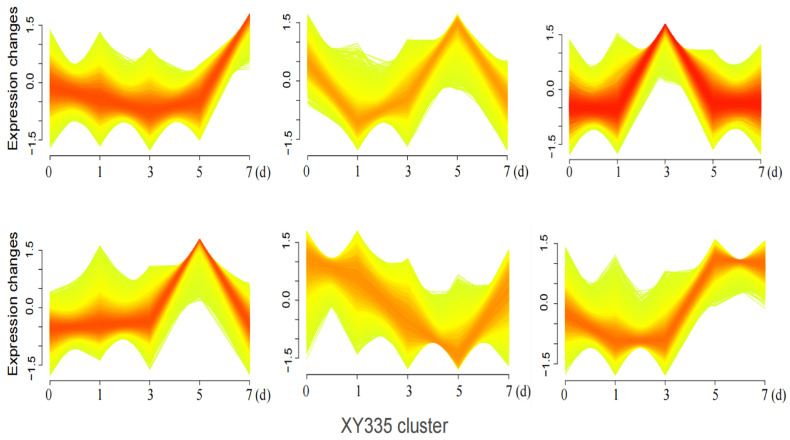
DEGs of XY335 were categorized into six clusters based on Mfuzz analysis under heat stress. The horizontal coordinates represent the different treatment times; the vertical coordinates represent the expression changes in these genes.

**Figure 8:**
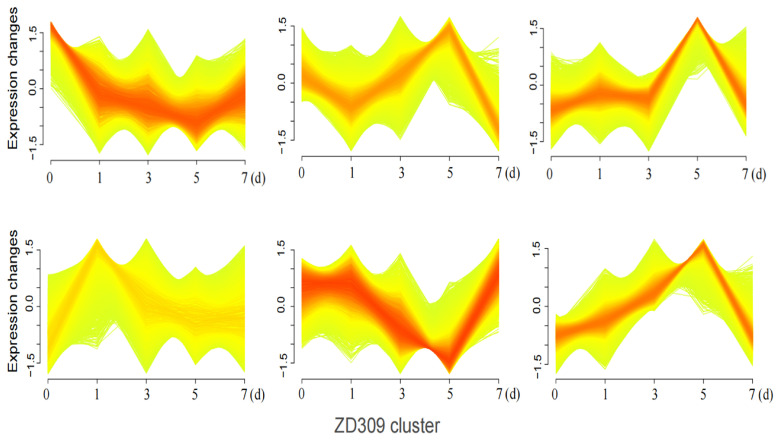
DEGs of ZD309 were categorized into six clusters based on Mfuzz analysis under heat stress. The horizontal coordinates represent the different treatment times; the vertical coordinates represent the expression changes in these genes.

**Figure 9:**
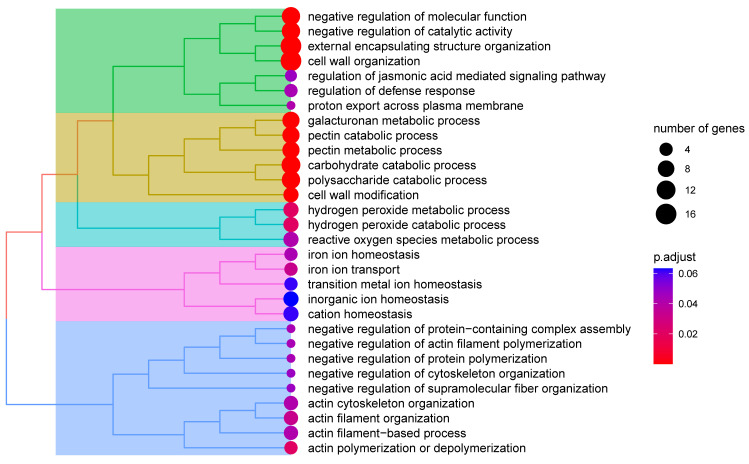
GO analysis of major DEGs under heat stress.

**Figure 10:**
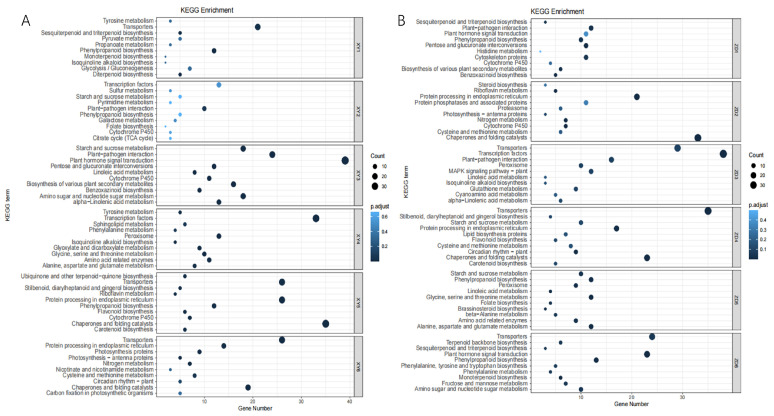
KEGG analysis of each cluster of XY335 and ZD309 under heat stress. (**A**) KEGG analysis of each cluster of XY335. (**B**) KEGG analysis of each cluster of ZD309.

The Mfuzz time-series analysis, based on the Fuzzy C-Means (FCM) algorithm 12, clusters genes with similar expression patterns to infer their potential involvement in shared biological processes or pathways. In this study, DEGs between the heat-sensitive variety XY335 and the heat-tolerant variety ZD309 were analyzed to investigate their dynamic responses to prolonged high-temperature treatment. These genes were divided into six clusters using Mfuzz (Figure 7 and Figure 8). In Cluster 3, DEGs exhibited a significant expression peak in XY335 on day 3 of high-temperature treatment, but no such peak was observed in ZD309. Further analysis revealed that these DEGs in ZD309 were predominantly clustered in Cluster 4, with expression peaking on day 1 of treatment. This indicated that the transcriptional response speed of the heat-tolerant variety is faster than that of the heat-sensitive variety. GO enrichment analysis (Figure 9) highlighted that these DEGs were primarily associated with: acid catalytic activity, pectin and galacturonan modification, hydrogen peroxide and ROS metabolism, and carbohydrate catabolic processes. The rapid transcriptional activation observed in ZD309 suggests that this enhanced regulatory mechanisms for mitigating oxidative stress and maintaining cell wall integrity under heat stress. KEGG pathway analysis of the six clusters revealed distinct functional enrichment patterns between XY335 and ZD309 under heat stress: In XY335, DEGs exhibited significant enrichment in specific pathways, notably including starch and sucrose metabolism, plant hormone signal transduction, and protein processing in the endoplasmic reticulum. For ZD309, DEGs were primarily enriched within metabolic and functional categories such as starch and sucrose metabolism, flavonoid biosynthesis, phenylalanine metabolism, and photosynthesis-antenna proteins (Figure 10). Notably, starch and sucrose metabolism exhibited significant enrichment in both varieties, suggesting its pivotal role in maize thermotolerance. This conserved pathway may regulate carbon allocation and energy homeostasis during heat adaptation, potentially by modulating starch synthesis and degradation dynamics.

### 3.5. Transcription Factor (TF) and Hormone Analysis

**Figure 11:**
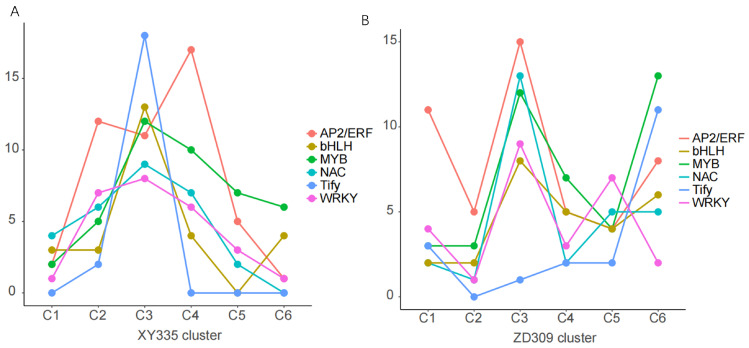
TFs analysis of Y335 and ZD309 based on Mfuzz under heat stress. (**A**) TFs analysis of XY335. (**B**) TFs analysis of ZD309. The X axis represents the six clusters divided by Mfuzz, while the Y axis represents the number of transcription factors. Each TF is represented by a different color.

TFs serve as pivotal regulators in diverse biological pathways. This study primarily identified six classes of transcription factors: AP2/ERF, bHLH, MYB, NAC, Tify, and WRKY. This research revealed that these transcription factors exhibited the highest numbers in the third cluster of XY335 and ZD309, respectively. This indicates that the expression patterns of most of the transcription factors in XY335 are similar to those in the third cluster of XY335, while those in ZD309 are predominantly aligned with the expression patterns observed in the third cluster of ZD309 (Figure 11).

**Figure 12:**
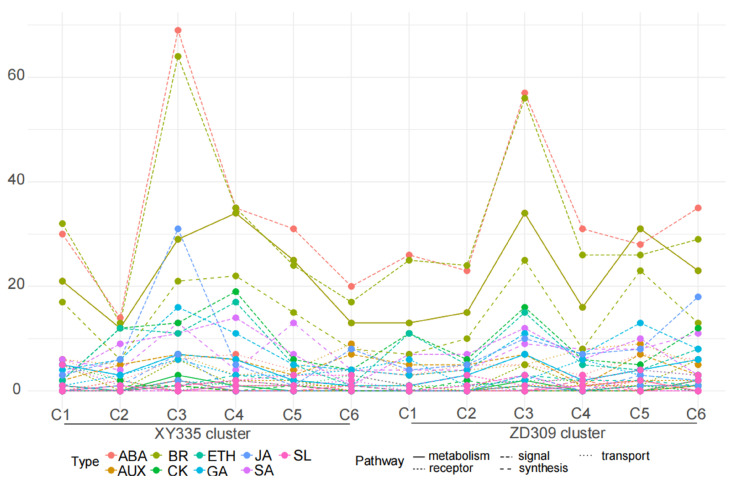
Distribution of phytohormone pathway-associated genes across different clusters based on Mfuzz under heat stress. The X axis represents six clusters of XY335 and ZD309, while the Y axis represents the number of DEGs for various hormones. Category 1 indicates different types of hormones, and Category 2 represents the respective functions of these hormones.

Plant hormones exert a critical regulatory effect on abiotic stress responses. Analysis of plant-hormone-related DEGs demonstrated that ABA (abscisic acid) and BR (brassinosteroid)-associated genes were the most abundant and were enriched in the third cluster of XY335 and ZD309 (Figure 12).

### 3.6. WGCNA Analysis

**Figure 13:**
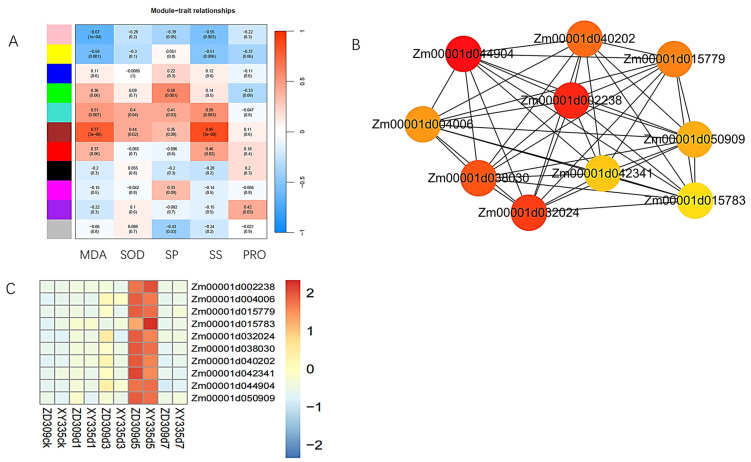
WGCNA analysis of XY335 and ZD309 under heat stress. (**A**) Joint analysis of physiological indicators and transcriptome data. (**B**) Interaction network of the top 10 genes with the highest weights, the colors of different circles represent the magnitudes of the weights. (**C**) Heatmap of the genes with the highest weights.

WGCNA can assess correlations among different modules to identify shared regulatory networks and biological pathways. In this study, an integrated analysis of physiological indicators and transcriptomic data revealed that the brown module exhibited the strongest associations with these modules, while the pink module showed negative correlations with all tested physiological indicators (Figure 13A).

Genes within the brown module were further analyzed using the Maximal Clique Centrality (MCC) method. The top 10 genes with the highest connectivity weights were selected to construct an interaction network and heatmap (Figure 13B,C), demonstrating that their expression peaked on the fifth day of high-temperature treatment. GO analysis of these 10 genes indicated that they functioned primarily in ubiquitin thiolesterase, RNA modification, protein domain specific binding and related processes, suggesting their critical roles in maintaining cellular homeostasis under heat stress (Table 1).

## 4. Discussion

High temperature, one of the most prevalent abiotic stresses, significantly inhibits plant growth and development, thereby leading to a substantial decrease in crop yields nationwide [30]. Under heat stress, plants experience the overproduction of ROS in specific cellular compartments, including chloroplasts, mitochondria, plasma membranes, peroxisomes, the apoplast, and the endoplasmic reticulum [31]. The excessive accumulation of ROS can disrupt multiple physiological processes, including photosynthesis, respiration, transpiration, membrane thermal stability, and osmotic regulation [32]. Thermosensitivity varies significantly across distinct developmental stages in maize. The reproductive phase, particularly anthesis, represents a critical growth period where heat stress exposure can cause substantial yield reductions [16]. Pollen formation, pollen development, and tassel development exhibit high sensitivity to heat stress [33,34,35]. Different maize genotypes exhibit distinct thermotolerance [19,36]. Comparing the response patterns of heat-tolerant and heat-sensitive maize under high-temperature stress aids in the elucidation of the molecular mechanisms governing maize heat tolerance.

High-temperature stress generally induces oxidative stress, leading to the peroxidation of membrane lipids and pigments [37]. This can be seen from the generation rate of superoxide anions and the content of MDA (Figure 1) in our study, which is consistent with the results of previous studies [38]. Our analysis revealed that DEGs associated with oxidoreductase activity and redox processes were significantly enriched (Figure 3). SOD is a key antioxidant enzyme that plays a crucial role in protecting plants from oxidative damage under various abiotic stresses, including high-temperature stress. High temperatures can lead to increased production of ROS, causing oxidative stress and damage to cellular components. SOD catalyzes the conversion of superoxide radicals (O_2_^−^) into less toxic molecules like hydrogen peroxide (H_2_O_2_) and molecular oxygen (O_2_), thereby mitigating ROS toxicity. In previous research, the expression of several SOD genes in maize was significantly upregulated under stress conditions, suggesting their involvement in stress response mechanisms [39]. The presence of stress-responsive cis-elements in the promoters of SOD genes, such as ABRE and DRE, indicates that these genes could be regulated by heat stress signaling pathways. Therefore, SODs likely contribute to heat tolerance in maize by maintaining ROS homeostasis and protecting cellular integrity under high-temperature stress. DEGs were also enriched in additional metabolic pathways, including secondary metabolite biosynthesis, transcriptional regulation, circadian rhythm, signal transduction, and amino acid metabolism (e.g., glutathione-related pathways), which is consistent with previous research [40,41,42]. Secondary metabolites play vital roles in plant adaptation and response to adverse environments [43]. Transcriptome analysis of seedlings from the maize inbred line B73 also revealed that pathways related to secondary metabolite biosynthesis, protein processing in the endoplasmic reticulum, and sucrose and starch metabolism may serve as core components in maize responses to heat stress [44]. In this study, genes associated with phenylpropanoid biosynthesis and the photosynthesis-antenna protein pathway were significantly enriched (Figure 10A,B), indicating that these pathways may represent the most fundamental mechanisms underlying maize heat tolerance.

DEGs that consistently appeared and had highly significant expression changes in all treatment comparison groups were identified in two maize varieties. In XY335, five genes were found: Zm00001d000216 (Transporters), Zm00001d015821 (Pentose and glucuronate interconversions), Zm00001d012482 (MAPK signaling pathway-plant), Zm00001d033222 (Carotenoid biosynthesis), and Zm00001d031449 (α-Linolenic acid metabolism). In ZD309, four genes were found: Zm00001d028116 (MAPK signaling pathway-plant), Zm00001d002287 (Plant-pathogen interaction), Zm00001d011377 (Plant hormone signal transduction), and Zm00001d017821 (Transcription factors). Notably, Zm00001d012482 and Zm00001d028116 are both involved in the MAPK signaling pathway. Zhang et al. found that the MAPK signaling pathway plays a crucial role in maize’s salt tolerance [45]. Wu et al. discovered that overexpressing *ZmMAPK1* enhances Arabidopsis’ tolerance to high temperature and drought [46]. In this study, genes involved in the MAPK signaling pathway in both ZD309 and XY335 were significantly upregulated following heat stress. Thus, it is hypothesized that these genes are associated with thermotolerance in maize.

Among these key genes, Zm00001d012482 is a homolog of wheat *TaWRKY24*. Overexpressing *TaWRKY24* in tobacco enhanced its drought and salt stress tolerance [47]. Zm00001d011377 is a homolog of *JAR1*, a GH3 family enzyme crucial for jasmonic acid (JA) biosynthesis via catalyzing JA to JA-Ile. Increased JA-Ile from *JAR1* improved Arabidopsis’ drought tolerance [48]. Zm00001d031449 is a homolog of lipoxygenase (LOX), a non-heme iron dioxygenase important for plant growth, development, and defense. Overexpressing pepper *CaLOX1* in Arabidopsis enhanced drought and salt tolerance by quickly scavenging ROS and inducing ABA biosynthesis-related genes [49]. In tomatoes, overexpressing *TomLoxD* showed resistance to leaf mold and high temperature [50].

Previous research has demonstrated that seven hormones, including ABA, auxin, JA, cytokinins (CKs), ethylene, gibberellin, and brassinosteroids (BR), may be implicated in the response to heat stress [40]. ABA alleviates heat-stress-induced ROS accumulation and oxidative damage by activating antioxidant enzyme systems including SOD, CAT, and POD [51]. Additionally, ABA regulates the expression of small heat shock proteins (sHSPs) and HSP70/90 to stabilize protein structures and prevent protein aggregation under high-temperature conditions [52]. In maize, ABA-mediated upregulation of HSP26 protects chloroplast proteins and maintains photosynthetic efficiency [31]. Similarly, BR reduces oxidative stress by enhancing the activities of SOD, CAT, and ascorbate peroxidase (APX) [53]. For instance, BR-treated tomato plants showed significantly decreased H_2_O_2_ levels and reduced lipid peroxidation under heat stress [54]. This study also found a large number of genes related to ABA and BR, indicating that these hormones are involved in the response to heat stress.

TFs play a pivotal role in plant responses to diverse abiotic and biotic stresses. Previous investigations have identified key families of TFs—including WRKY, NAC, MYB, AP2/ERF and b-ZIP—which have been demonstrated to mediate plant adaptation to various stress conditions [55,56,57,58]. WRKY, including WRKY25 and WRKY33, mitigate heat-induced oxidative damage by regulating antioxidant enzyme genes such as *APX1* and *CAT2*. Transgenic Arabidopsis plants with *WRKY25* overexpression demonstrated increased superoxide dismutase activity and enhanced heat tolerance capacity [59]. The NAC family serves as a crucial regulator integrating stress adaptation with developmental processes. Specific members (*ANAC019*, *ANAC055*) enhance thermotolerance through transcriptional activation of HSPs and ROS-scavenging genes such as *GST6* [60]. Other NACs, like *NTL4*, suppress growth-related genes under heat stress to balance stress responses and development [61]. Additionally, bZIPs such as bZIP28 and bZIP60 function as endoplasmic reticulum (ER) stress sensors that activate heat stress responses through the unfolded protein response (UPR) pathway [62]. In this study, transcription factor families such as WRKY, b-ZIP, NAC and MYB were identified, and these genes might be critically involved in maize’s adaptation to high-temperature stress.

## 5. Conclusions

The flowering stage is recognized as one of the most sensitive periods in maize with respect to heat stress. Such stress during this period can damage pollen germination, increase the risk of blighted grains, lower the seed-setting rate and grain weight, and eventually lead to significant yield losses. In this study, two maize varieties (one heat-tolerant variety and one heat-sensitive variety) were used to uncover the heat resistance mechanism by transcriptome analysis during the flowering stage. The results showed that in both varieties, DEGs mainly clustered on the fifth day of high-temperature treatment, with the heat-tolerant variety ZD309 presenting more DEGs than the heat-sensitive variety XY335. The analysis of physiological indexes also showed that the activity of SOD, the content of PRO, MDA, soluble sugar, and soluble protein increased significantly after heat treatment, and with the highest content on the fifth day. By integrating transcriptome data analysis with physiological indicators, we found that some key genes were predominantly active on the fifth day. The most significant changes DEGs in XY335 were mainly concentrated in galactose metabolism, pectin catabolism and carbohydrate catabolism. The GO function annotation results showed that target genes selected by WGCNA were enriched in ubiquitin thiolesterase, RNA modification, protein-domain-specific binding and other aspects. This suggests their primary role at the protein level. Overall, our findings advance the understanding of the molecular mechanisms governing maize adaptation to high-temperature stress.

## Figures and Tables

**Table 1:** A total of 10 genes with the highest weights were screened, which showed high expression levels (fold change > 2) under heat stress.

GeneID	Description
Zm00001d002238	Ubiquitin thiolesterase
Zm00001d044904	- PPR repeat (PPR)
Zm00001d042341	RNA modification
Zm00001d015779	14-3-3 protein
Zm00001d032024	MYB-like DNA-binding protein
Zm00001d040202	A.THALIANA mRNA (ORF19) from chromosome III
Zm00001d015783	Glucan endo-1,3-β-D-glucosidase/Laminarinase
Zm00001d050909	-
Zm00001d004006	- AMP-activated protein kinase
Zm00001d038030	Protein of unknown function (DUF2985)

## Data Availability

The datasets supporting the conclusions of this article are included within this article (and its additional files). The sequencing database for maize can be downloaded from NCBI under the accession number PRJNA1235981, and the data will be shared on reasonable request of the corresponding author.
